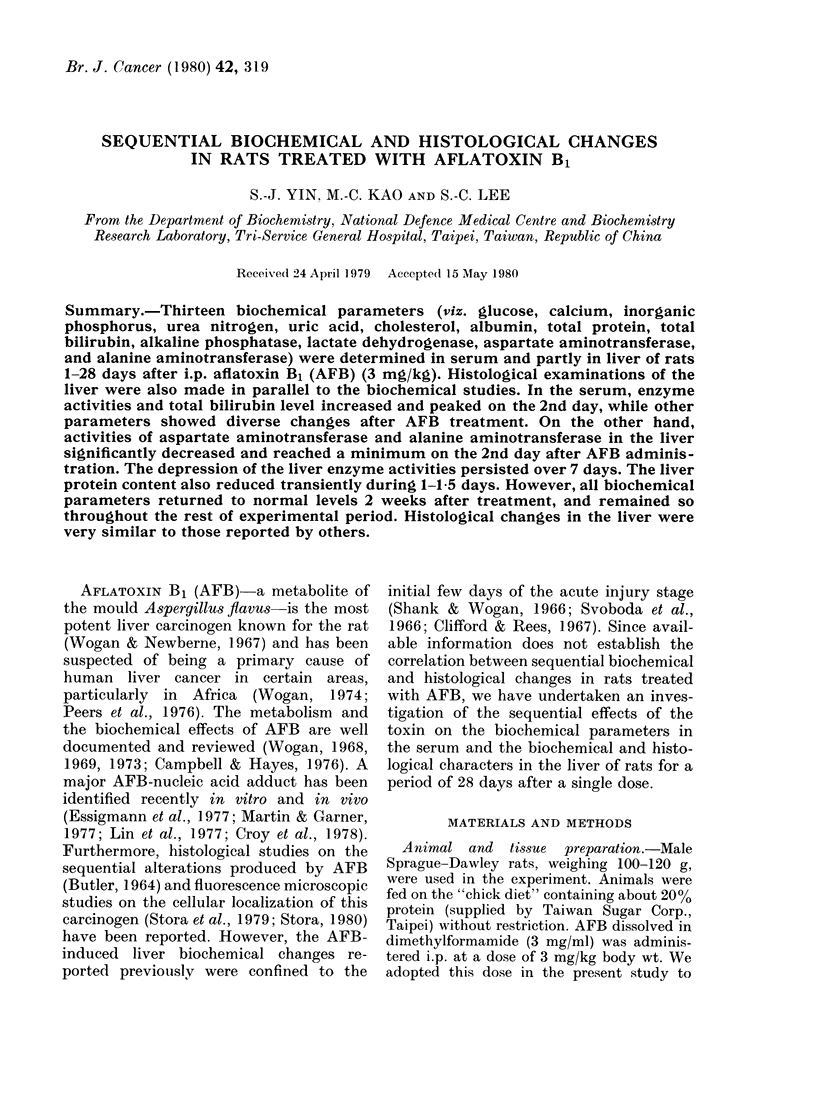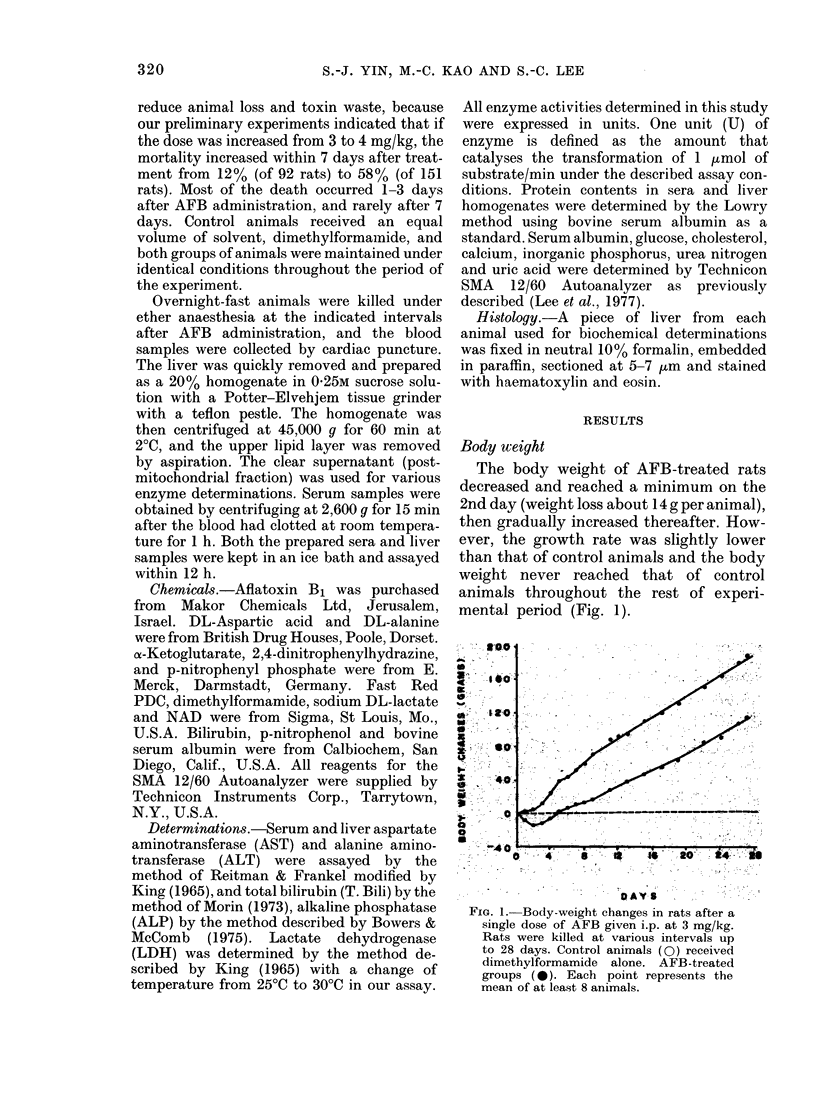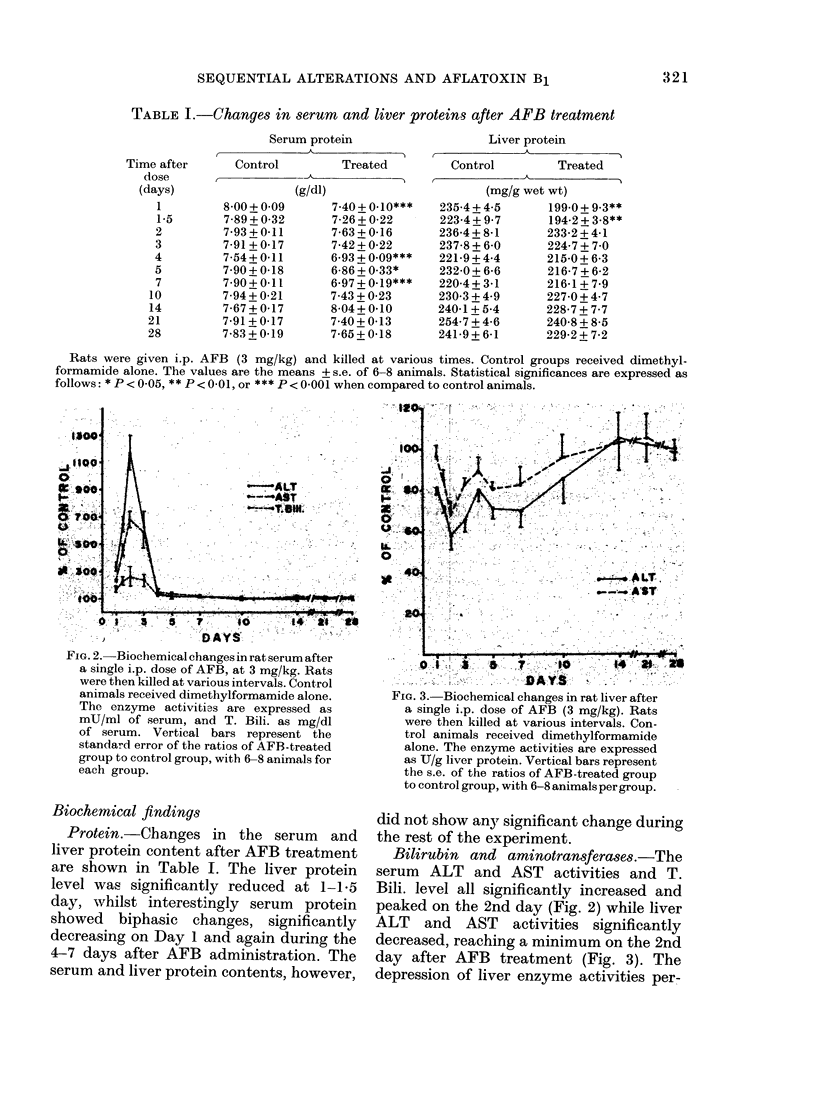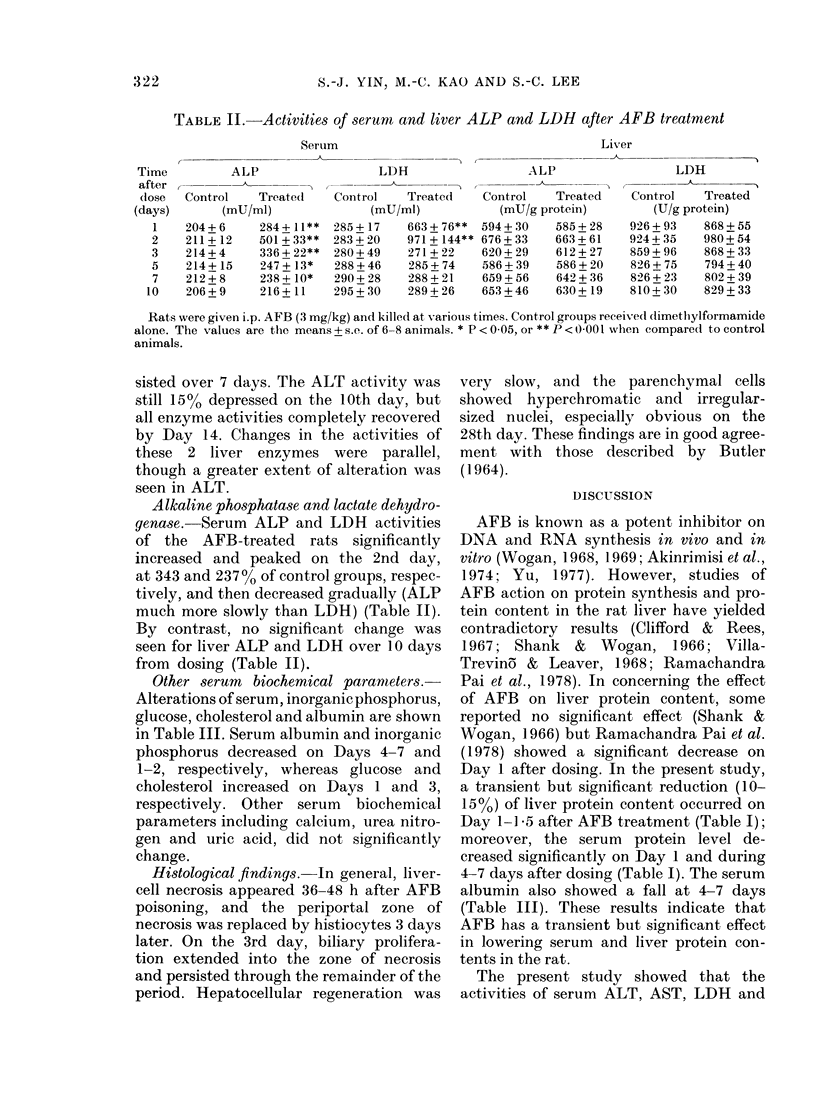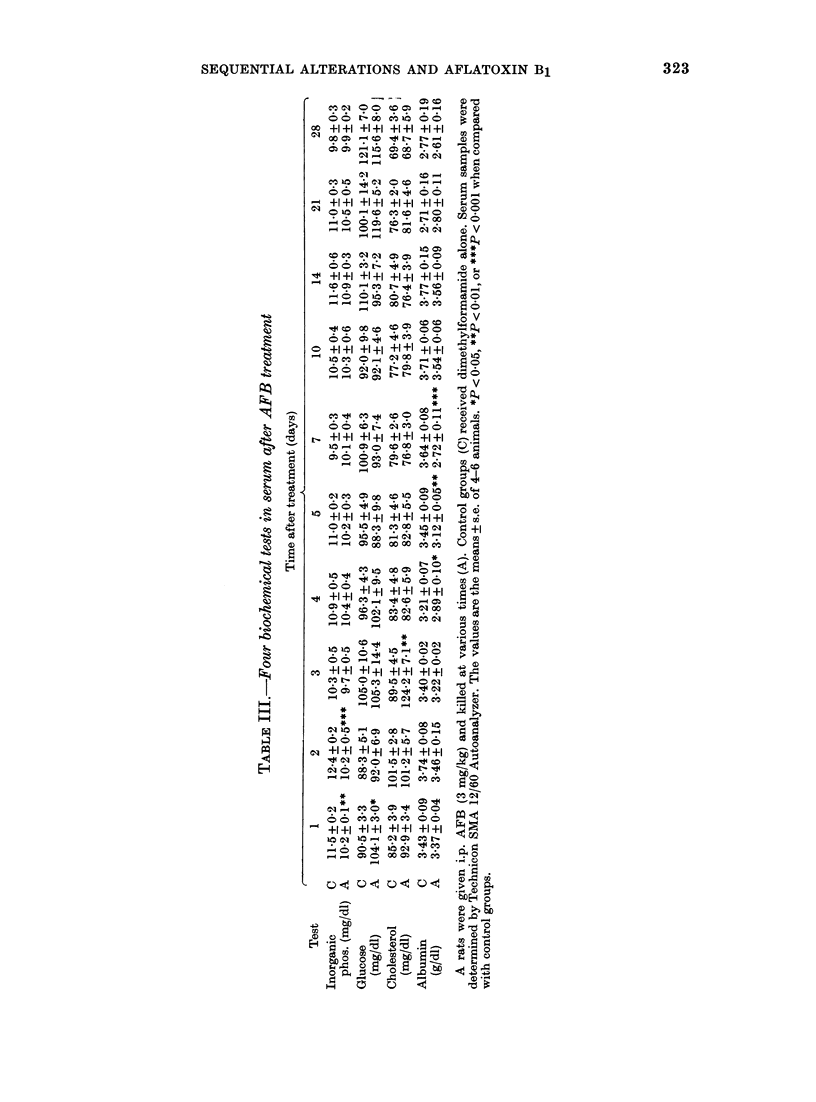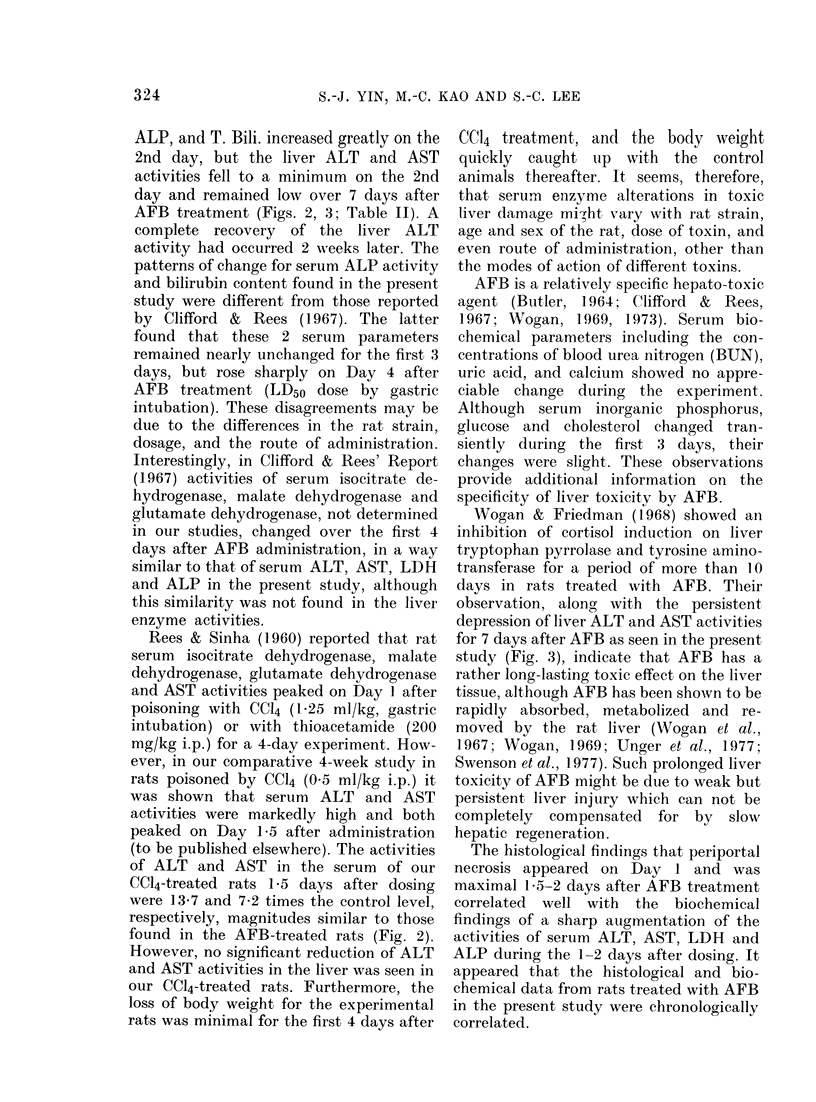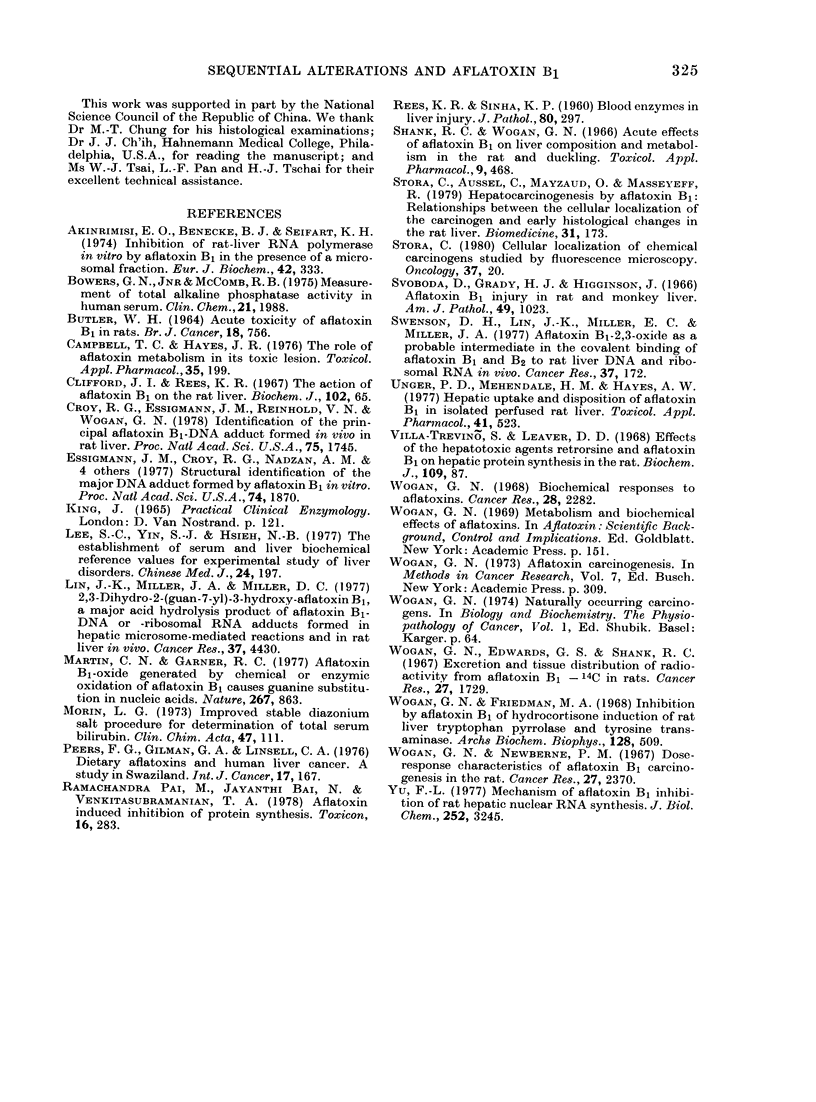# Sequential biochemical and histological changes in rats treated with aflatoxin B1.

**DOI:** 10.1038/bjc.1980.233

**Published:** 1980-08

**Authors:** S. J. Yin, M. C. Kao, S. C. Lee

## Abstract

Thirteen biochemical parameters (viz. glucose, calcium, inorganic phosphorous, urea nitrogen, uric acid, cholesterol, albumin, total protein, total bilirubin, alkaline phosphatase, lactate dehydrogenase, aspartate aminotransferase, and alanine aminotransferase) were determined in serum and partly in liver of rats 1-28 days after i.p. aflatoxin B1 (AFB) (3 mg/kg). Histological examinations of the liver were also made in parallel to the biochemical studies. In the serum, enzyme activities and total bilirubin level increased and peaked on the 2nd day, while other activities of aspartate aminotransferase and alanine aminotransferase in the liver significantly decreased and reached a minimum on the 2nd day after AFB administration. The depression of the liver enzyme activities persisted over 7 days. The liver protein content also reduced transiently during 1-1.5 days. However, all biochemical parameters returned to normal levels 2 weeks after treatment, and remained so throughout the rest of experimental period. Histological changes in the liver were very similar to those reported by other.


*Br. J. Cancer* (1980) **42**, 319

### SEQUENTIAL BIOCHEMICAL AND HISTOLOGICAL CHANGES IN RATS TREATED WITH AFLATOXIN $B_1$

S.-J. YIN, M.-C. KAO AND S.-C. LEE

*From the Department of Biochemistry, National Defence Medical Centre and Biochemistry Research Laboratory, Tri-Service General Hospital, Taipei, Taiwan, Republic of China*

Received 24 April 1979 Accepted 15 May 1980

**Summary.—Thirteen biochemical parameters (*viz.* glucose, calcium, inorganic phosphorus, urea nitrogen, uric acid, cholesterol, albumin, total protein, total bilirubin, alkaline phosphatase, lactate dehydrogenase, aspartate aminotransferase, and alanine aminotransferase) were determined in serum and partly in liver of rats 1–28 days after i.p. aflatoxin $B_1$ (AFB) (3 mg/kg). Histological examinations of the liver were also made in parallel to the biochemical studies. In the serum, enzyme activities and total bilirubin level increased and peaked on the 2nd day, while other parameters showed diverse changes after AFB treatment. On the other hand, activities of aspartate aminotransferase and alanine aminotransferase in the liver significantly decreased and reached a minimum on the 2nd day after AFB administration. The depression of the liver enzyme activities persisted over 7 days. The liver protein content also reduced transiently during 1–1·5 days. However, all biochemical parameters returned to normal levels 2 weeks after treatment, and remained so throughout the rest of experimental period. Histological changes in the liver were very similar to those reported by others.**

AFLATOXIN $B_1$ (AFB)—a metabolite of the mould *Aspergillus flavus*—is the most potent liver carcinogen known for the rat (Wogan & Newberne, 1967) and has been suspected of being a primary cause of human liver cancer in certain areas, particularly in Africa (Wogan, 1974; Peers *et al.*, 1976). The metabolism and the biochemical effects of AFB are well documented and reviewed (Wogan, 1968, 1969, 1973; Campbell & Hayes, 1976). A major AFB-nucleic acid adduct has been identified recently *in vitro* and *in vivo* (Essigmann *et al.*, 1977; Martin & Garner, 1977; Lin *et al.*, 1977; Croy *et al.*, 1978). Furthermore, histological studies on the sequential alterations produced by AFB (Butler, 1964) and fluorescence microscopic studies on the cellular localization of this carcinogen (Stora *et al.*, 1979; Stora, 1980) have been reported. However, the AFB-induced liver biochemical changes reported previously were confined to the initial few days of the acute injury stage (Shank & Wogan, 1966; Svoboda *et al.*, 1966; Clifford & Rees, 1967). Since available information does not establish the correlation between sequential biochemical and histological changes in rats treated with AFB, we have undertaken an investigation of the sequential effects of the toxin on the biochemical parameters in the serum and the biochemical and histological characters in the liver of rats for a period of 28 days after a single dose.

### MATERIALS AND METHODS

*Animal and tissue preparation.*—Male Sprague–Dawley rats, weighing 100–120 g, were used in the experiment. Animals were fed on the "chick diet" containing about 20% protein (supplied by Taiwan Sugar Corp., Taipei) without restriction. AFB dissolved in dimethylformamide (3 mg/ml) was administered i.p. at a dose of 3 mg/kg body wt. We adopted this dose in the present study to reduce animal loss and toxin waste, because our preliminary experiments indicated that if the dose was increased from 3 to 4 mg/kg, the mortality increased within 7 days after treatment from 12% (of 92 rats) to 58% (of 151 rats). Most of the death occurred 1–3 days after AFB administration, and rarely after 7 days. Control animals received an equal volume of solvent, dimethylformamide, and both groups of animals were maintained under identical conditions throughout the period of the experiment.

Overnight-fast animals were killed under ether anaesthesia at the indicated intervals after AFB administration, and the blood samples were collected by cardiac puncture. The liver was quickly removed and prepared as a 20% homogenate in 0·25M sucrose solution with a Potter–Elvehjem tissue grinder with a teflon pestle. The homogenate was then centrifuged at 45,000 *g* for 60 min at 2°C, and the upper lipid layer was removed by aspiration. The clear supernatant (post-mitochondrial fraction) was used for various enzyme determinations. Serum samples were obtained by centrifuging at 2,600 *g* for 15 min after the blood had clotted at room temperature for 1 h. Both the prepared sera and liver samples were kept in an ice bath and assayed within 12 h.

*Chemicals.*—Aflatoxin $B_1$ was purchased from Makor Chemicals Ltd, Jerusalem, Israel. DL-Aspartic acid and DL-alanine were from British Drug Houses, Poole, Dorset. α-Ketoglutarate, 2,4-dinitrophenylhydrazine, and p-nitrophenyl phosphate were from E. Merck, Darmstadt, Germany. Fast Red PDC, dimethylformamide, sodium DL-lactate and NAD were from Sigma, St Louis, Mo., U.S.A. Bilirubin, p-nitrophenol and bovine serum albumin were from Calbiochem, San Diego, Calif., U.S.A. All reagents for the SMA 12/60 Autoanalyzer were supplied by Technicon Instruments Corp., Tarrytown, N.Y., U.S.A.

*Determinations.*—Serum and liver aspartate aminotransferase (AST) and alanine aminotransferase (ALT) were assayed by the method of Reitman & Frankel modified by King (1965), and total bilirubin (T. Bili) by the method of Morin (1973), alkaline phosphatase (ALP) by the method described by Bowers & McComb (1975). Lactate dehydrogenase (LDH) was determined by the method described by King (1965) with a change of temperature from 25°C to 30°C in our assay. All enzyme activities determined in this study were expressed in units. One unit (U) of enzyme is defined as the amount that catalyses the transformation of 1 μmol of substrate/min under the described assay conditions. Protein contents in sera and liver homogenates were determined by the Lowry method using bovine serum albumin as a standard. Serum albumin, glucose, cholesterol, calcium, inorganic phosphorus, urea nitrogen and uric acid were determined by Technicon SMA 12/60 Autoanalyzer as previously described (Lee *et al.*, 1977).

*Histology.*—A piece of liver from each animal used for biochemical determinations was fixed in neutral 10% formalin, embedded in paraffin, sectioned at 5–7 μm and stained with haematoxylin and eosin.

### RESULTS

### *Body weight*

The body weight of AFB-treated rats decreased and reached a minimum on the 2nd day (weight loss about 14 g per animal), then gradually increased thereafter. However, the growth rate was slightly lower than that of control animals and the body weight never reached that of control animals throughout the rest of experimental period (Fig. 1).

Fig. 1.—Body-weight changes in rats after a single dose of AFB given i.p. at 3 mg/kg. Rats were killed at various intervals up to 28 days. Control animals (○) received dimethylformamide alone. AFB-treated groups (●). Each point represents the mean of at least 8 animals.

TABLE I.—*Changes in serum and liver proteins after AFB treatment*

| Time after dose (days) | Serum protein Control (g/dl) | Serum protein Treated (g/dl) | Liver protein Control (mg/g wet wt) | Liver protein Treated (mg/g wet wt) |
|---|---|---|---|---|
| 1 | 8·00 ± 0·09 | 7·40 ± 0·10*** | 235·4 ± 4·5 | 199·0 ± 9·3** |
| 1·5 | 7·89 ± 0·32 | 7·26 ± 0·22 | 223·4 ± 9·7 | 194·2 ± 3·8** |
| 2 | 7·93 ± 0·11 | 7·63 ± 0·16 | 236·4 ± 8·1 | 233·2 ± 4·1 |
| 3 | 7·91 ± 0·17 | 7·42 ± 0·22 | 237·8 ± 6·0 | 224·7 ± 7·0 |
| 4 | 7·54 ± 0·11 | 6·93 ± 0·09*** | 221·9 ± 4·4 | 215·0 ± 6·3 |
| 5 | 7·90 ± 0·18 | 6·86 ± 0·33* | 232·0 ± 6·6 | 216·7 ± 6·2 |
| 7 | 7·90 ± 0·11 | 6·97 ± 0·19*** | 220·4 ± 3·1 | 216·1 ± 7·9 |
| 10 | 7·94 ± 0·21 | 7·43 ± 0·23 | 230·3 ± 4·9 | 227·0 ± 4·7 |
| 14 | 7·67 ± 0·17 | 8·04 ± 0·10 | 240·1 ± 5·4 | 228·7 ± 7·7 |
| 21 | 7·91 ± 0·17 | 7·40 ± 0·13 | 254·7 ± 4·6 | 240·8 ± 8·5 |
| 28 | 7·83 ± 0·19 | 7·65 ± 0·18 | 241·9 ± 6·1 | 229·2 ± 7·2 |

Rats were given i.p. AFB (3 mg/kg) and killed at various times. Control groups received dimethylformamide alone. The values are the means ± s.e. of 6–8 animals. Statistical significances are expressed as follows: * $P < 0{\cdot}05$, ** $P < 0{\cdot}01$, or *** $P < 0{\cdot}001$ when compared to control animals.

FIG. 2.—Biochemical changes in rat serum after a single i.p. dose of AFB, at 3 mg/kg. Rats were then killed at various intervals. Control animals received dimethylformamide alone. The enzyme activities are expressed as mU/ml of serum, and T. Bili. as mg/dl of serum. Vertical bars represent the standard error of the ratios of AFB-treated group to control group, with 6–8 animals for each group.

FIG. 3.—Biochemical changes in rat liver after a single i.p. dose of AFB (3 mg/kg). Rats were then killed at various intervals. Control animals received dimethylformamide alone. The enzyme activities are expressed as U/g liver protein. Vertical bars represent the s.e. of the ratios of AFB-treated group to control group, with 6–8 animals per group.

*Biochemical findings*

*Protein.*—Changes in the serum and liver protein content after AFB treatment are shown in Table I. The liver protein level was significantly reduced at 1–1·5 day, whilst interestingly serum protein showed biphasic changes, significantly decreasing on Day 1 and again during the 4–7 days after AFB administration. The serum and liver protein contents, however, did not show any significant change during the rest of the experiment.

*Bilirubin and aminotransferases.*—The serum ALT and AST activities and T. Bili. level all significantly increased and peaked on the 2nd day (Fig. 2) while liver ALT and AST activities significantly decreased, reaching a minimum on the 2nd day after AFB treatment (Fig. 3). The depression of liver enzyme activities per-

TABLE II.—*Activities of serum and liver ALP and LDH after AFB treatment*

| Time after dose (days) | Serum ALP Control (mU/ml) | Serum ALP Treated (mU/ml) | Serum LDH Control (mU/ml) | Serum LDH Treated (mU/ml) | Liver ALP Control (mU/g protein) | Liver ALP Treated (mU/g protein) | Liver LDH Control (U/g protein) | Liver LDH Treated (U/g protein) |
|---|---|---|---|---|---|---|---|---|
| 1 | 204±6 | 284±11** | 285±17 | 663±76** | 594±30 | 585±28 | 926±93 | 868±55 |
| 2 | 211±12 | 501±33** | 283±20 | 971±144** | 676±33 | 663±61 | 924±35 | 980±54 |
| 3 | 214±4 | 336±22** | 280±49 | 271±22 | 620±29 | 612±27 | 859±96 | 868±33 |
| 5 | 214±15 | 247±13* | 288±46 | 285±74 | 586±39 | 586±20 | 826±75 | 794±40 |
| 7 | 212±8 | 238±10* | 290±28 | 288±21 | 659±56 | 642±36 | 826±23 | 802±39 |
| 10 | 206±9 | 216±11 | 295±30 | 289±26 | 653±46 | 630±19 | 810±30 | 829±33 |

Rats were given i.p. AFB (3 mg/kg) and killed at various times. Control groups received dimethylformamide alone. The values are the means ± s.e. of 6–8 animals. * P < 0·05, or ** $P$ < 0·001 when compared to control animals.

sisted over 7 days. The ALT activity was still 15% depressed on the 10th day, but all enzyme activities completely recovered by Day 14. Changes in the activities of these 2 liver enzymes were parallel, though a greater extent of alteration was seen in ALT.

*Alkaline phosphatase and lactate dehydrogenase.*—Serum ALP and LDH activities of the AFB-treated rats significantly increased and peaked on the 2nd day, at 343 and 237% of control groups, respectively, and then decreased gradually (ALP much more slowly than LDH) (Table II). By contrast, no significant change was seen for liver ALP and LDH over 10 days from dosing (Table II).

*Other serum biochemical parameters.*—Alterations of serum, inorganic phosphorus, glucose, cholesterol and albumin are shown in Table III. Serum albumin and inorganic phosphorus decreased on Days 4–7 and 1–2, respectively, whereas glucose and cholesterol increased on Days 1 and 3, respectively. Other serum biochemical parameters including calcium, urea nitrogen and uric acid, did not significantly change.

*Histological findings.*—In general, liver-cell necrosis appeared 36–48 h after AFB poisoning, and the periportal zone of necrosis was replaced by histiocytes 3 days later. On the 3rd day, biliary proliferation extended into the zone of necrosis and persisted through the remainder of the period. Hepatocellular regeneration was very slow, and the parenchymal cells showed hyperchromatic and irregular-sized nuclei, especially obvious on the 28th day. These findings are in good agreement with those described by Butler (1964).

### DISCUSSION

AFB is known as a potent inhibitor on DNA and RNA synthesis *in vivo* and *in vitro* (Wogan, 1968, 1969; Akinrimisi *et al.*, 1974; Yu, 1977). However, studies of AFB action on protein synthesis and protein content in the rat liver have yielded contradictory results (Clifford & Rees, 1967; Shank & Wogan, 1966; Villa-Trevinõ & Leaver, 1968; Ramachandra Pai *et al.*, 1978). In concerning the effect of AFB on liver protein content, some reported no significant effect (Shank & Wogan, 1966) but Ramachandra Pai *et al.* (1978) showed a significant decrease on Day 1 after dosing. In the present study, a transient but significant reduction (10–15%) of liver protein content occurred on Day 1–1·5 after AFB treatment (Table I); moreover, the serum protein level decreased significantly on Day 1 and during 4–7 days after dosing (Table I). The serum albumin also showed a fall at 4–7 days (Table III). These results indicate that AFB has a transient but significant effect in lowering serum and liver protein contents in the rat.

The present study showed that the activities of serum ALT, AST, LDH and

TABLE III.—*Four biochemical tests in serum after AFB treatment*

| Test | | Time after treatment (days) 1 | 2 | 3 | 4 | 5 | 7 | 10 | 14 | 21 | 28 |
|---|---|---|---|---|---|---|---|---|---|---|---|
| Inorganic | C | 11·5±0·2 | 12·4±0·2 | 10·3±0·5 | 10·9±0·5 | 11·0±0·2 | 9·5±0·3 | 10·5±0·4 | 11·6±0·6 | 11·0±0·3 | 9·8±0·3 |
| phos. (mg/dl) | A | 10·2±0·1** | 10·2±0·5*** | 9·7±0·5 | 10·4±0·4 | 10·2±0·3 | 10·1±0·4 | 10·3±0·6 | 10·9±0·3 | 10·5±0·5 | 9·9±0·2 |
| Glucose | C | 90·5±3·3 | 88·3±5·1 | 105·0±10·6 | 96·3±4·3 | 95·5±4·9 | 100·9±6·3 | 92·0±9·8 | 110·1±3·2 | 100·1±14·2 | 121·1±7·0 |
| (mg/dl) | A | 104·1±3·0* | 92·0±6·9 | 105·3±14·4 | 102·1±9·5 | 88·3±9·8 | 93·0±7·4 | 92·1±4·6 | 95·3±7·2 | 119·6±5·2 | 115·6±8·0 |
| Cholesterol | C | 85·2±3·9 | 101·5±2·8 | 89·5±4·5 | 83·4±4·8 | 81·3±4·6 | 79·6±2·6 | 77·2±4·6 | 80·7±4·9 | 76·3±2·0 | 69·4±3·6 |
| (mg/dl) | A | 92·9±3·4 | 101·2±5·7 | 124·2±7·1** | 82·6±5·9 | 82·8±5·5 | 76·8±3·0 | 79·8±3·9 | 76·4±3·9 | 81·6±4·6 | 68·7±5·9 |
| Albumin | C | 3·43±0·09 | 3·74±0·08 | 3·40±0·02 | 3·21±0·07 | 3·45±0·09 | 3·64±0·08 | 3·71±0·06 | 3·77±0·15 | 2·71±0·16 | 2·77±0·19 |
| (g/dl) | A | 3·37±0·04 | 3·46±0·15 | 3·22±0·02 | 2·89±0·10* | 3·12±0·05** | 2·72±0·11*** | 3·54±0·06 | 3·56±0·09 | 2·80±0·11 | 2·61±0·16 |

A rats were given i.p. AFB (3 mg/kg) and killed at various times (A). Control groups (C) received dimethylformamide alone. Serum samples were determined by Technicon SMA 12/60 Autoanalyzer. The values are the means ± s.e. of 4–6 animals. $^{*}P < 0·05$, $^{**}P < 0·01$, or $^{***}P < 0·001$ when compared with control groups.

ALP, and T. Bili. increased greatly on the 2nd day, but the liver ALT and AST activities fell to a minimum on the 2nd day and remained low over 7 days after AFB treatment (Figs. 2, 3; Table II). A complete recovery of the liver ALT activity had occurred 2 weeks later. The patterns of change for serum ALP activity and bilirubin content found in the present study were different from those reported by Clifford & Rees (1967). The latter found that these 2 serum parameters remained nearly unchanged for the first 3 days, but rose sharply on Day 4 after AFB treatment ($LD_{50}$ dose by gastric intubation). These disagreements may be due to the differences in the rat strain, dosage, and the route of administration. Interestingly, in Clifford & Rees' Report (1967) activities of serum isocitrate dehydrogenase, malate dehydrogenase and glutamate dehydrogenase, not determined in our studies, changed over the first 4 days after AFB administration, in a way similar to that of serum ALT, AST, LDH and ALP in the present study, although this similarity was not found in the liver enzyme activities.

Rees & Sinha (1960) reported that rat serum isocitrate dehydrogenase, malate dehydrogenase, glutamate dehydrogenase and AST activities peaked on Day 1 after poisoning with $CCl_4$ (1·25 ml/kg, gastric intubation) or with thioacetamide (200 mg/kg i.p.) for a 4-day experiment. However, in our comparative 4-week study in rats poisoned by $CCl_4$ (0·5 ml/kg i.p.) it was shown that serum ALT and AST activities were markedly high and both peaked on Day 1·5 after administration (to be published elsewhere). The activities of ALT and AST in the serum of our $CCl_4$-treated rats 1·5 days after dosing were 13·7 and 7·2 times the control level, respectively, magnitudes similar to those found in the AFB-treated rats (Fig. 2). However, no significant reduction of ALT and AST activities in the liver was seen in our $CCl_4$-treated rats. Furthermore, the loss of body weight for the experimental rats was minimal for the first 4 days after $CCl_4$ treatment, and the body weight quickly caught up with the control animals thereafter. It seems, therefore, that serum enzyme alterations in toxic liver damage might vary with rat strain, age and sex of the rat, dose of toxin, and even route of administration, other than the modes of action of different toxins.

AFB is a relatively specific hepato-toxic agent (Butler, 1964; Clifford & Rees, 1967; Wogan, 1969, 1973). Serum biochemical parameters including the concentrations of blood urea nitrogen (BUN), uric acid, and calcium showed no appreciable change during the experiment. Although serum inorganic phosphorus, glucose and cholesterol changed transiently during the first 3 days, their changes were slight. These observations provide additional information on the specificity of liver toxicity by AFB.

Wogan & Friedman (1968) showed an inhibition of cortisol induction on liver tryptophan pyrrolase and tyrosine aminotransferase for a period of more than 10 days in rats treated with AFB. Their observation, along with the persistent depression of liver ALT and AST activities for 7 days after AFB as seen in the present study (Fig. 3), indicate that AFB has a rather long-lasting toxic effect on the liver tissue, although AFB has been shown to be rapidly absorbed, metabolized and removed by the rat liver (Wogan *et al.*, 1967; Wogan, 1969; Unger *et al.*, 1977; Swenson *et al.*, 1977). Such prolonged liver toxicity of AFB might be due to weak but persistent liver injury which can not be completely compensated for by slow hepatic regeneration.

The histological findings that periportal necrosis appeared on Day 1 and was maximal 1·5–2 days after AFB treatment correlated well with the biochemical findings of a sharp augmentation of the activities of serum ALT, AST, LDH and ALP during the 1–2 days after dosing. It appeared that the histological and biochemical data from rats treated with AFB in the present study were chronologically correlated.

This work was supported in part by the National Science Council of the Republic of China. We thank Dr M.-T. Chung for his histological examinations; Dr J. J. Ch'ih, Hahnemann Medical College, Philadelphia, U.S.A., for reading the manuscript; and Ms W.-J. Tsai, L.-F. Pan and H.-J. Tschai for their excellent technical assistance.

### REFERENCES

AKINRIMISI, E. O., BENECKE, B. J. & SEIFART, K. H. (1974) Inhibition of rat-liver RNA polymerase *in vitro* by aflatoxin $B_1$ in the presence of a microsomal fraction. *Eur. J. Biochem.*, **42**, 333.

BOWERS, G. N., JNR & McCOMB, R. B. (1975) Measurement of total alkaline phosphatase activity in human serum. *Clin. Chem.*, **21**, 1988.

BUTLER, W. H. (1964) Acute toxicity of aflatoxin $B_1$ in rats. *Br. J. Cancer*, **18**, 756.

CAMPBELL, T. C. & HAYES, J. R. (1976) The role of aflatoxin metabolism in its toxic lesion. *Toxicol. Appl. Pharmacol.*, **35**, 199.

CLIFFORD, J. I. & REES, K. R. (1967) The action of aflatoxin $B_1$ on the rat liver. *Biochem. J.*, **102**, 65.

CROY, R. G., ESSIGMANN, J. M., REINHOLD, V. N. & WOGAN, G. N. (1978) Identification of the principal aflatoxin $B_1$-DNA adduct formed *in vivo* in rat liver. *Proc. Natl Acad. Sci. U.S.A.*, **75**, 1745.

ESSIGMANN, J. M., CROY, R. G., NADZAN, A. M. & 4 others (1977) Structural identification of the major DNA adduct formed by aflatoxin $B_1$ *in vitro*. *Proc. Natl Acad. Sci. U.S.A.*, **74**, 1870.

KING, J. (1965) *Practical Clinical Enzymology.* London: D. Van Nostrand. p. 121.

LEE, S.-C., YIN, S.-J. & HSIEH, N.-B. (1977) The establishment of serum and liver biochemical reference values for experimental study of liver disorders. *Chinese Med. J.*, **24**, 197.

LIN, J.-K., MILLER, J. A. & MILLER, D. C. (1977) 2,3-Dihydro-2-(guan-7-yl)-3-hydroxy-aflatoxin $B_1$, a major acid hydrolysis product of aflatoxin $B_1$-DNA or -ribosomal RNA adducts formed in hepatic microsome-mediated reactions and in rat liver *in vivo*. *Cancer Res.*, **37**, 4430.

MARTIN, C. N. & GARNER, R. C. (1977) Aflatoxin $B_1$-oxide generated by chemical or enzymic oxidation of aflatoxin $B_1$ causes guanine substitution in nucleic acids. *Nature*, **267**, 863.

MORIN, L. G. (1973) Improved stable diazonium salt procedure for determination of total serum bilirubin. *Clin. Chim. Acta*, **47**, 111.

PEERS, F. G., GILMAN, G. A. & LINSELL, C. A. (1976) Dietary aflatoxins and human liver cancer. A study in Swaziland. *Int. J. Cancer*, **17**, 167.

RAMACHANDRA PAI, M., JAYANTHI BAI, N. & VENKITASUBRAMANIAN, T. A. (1978) Aflatoxin induced inhitibion of protein synthesis. *Toxicon*, **16**, 283.

REES, K. R. & SINHA, K. P. (1960) Blood enzymes in liver injury. *J. Pathol.*, **80**, 297.

SHANK, R. C. & WOGAN, G. N. (1966) Acute effects of aflatoxin $B_1$ on liver composition and metabolism in the rat and duckling. *Toxicol. Appl. Pharmacol.*, **9**, 468.

STORA, C., AUSSEL, C., MAYZAUD, O. & MASSEYEFF, R. (1979) Hepatocarcinogenesis by aflatoxin $B_1$: Relationships between the cellular localization of the carcinogen and early histological changes in the rat liver. *Biomedicine*, **31**, 173.

STORA, C. (1980) Cellular localization of chemical carcinogens studied by fluorescence microscopy. *Oncology*, **37**, 20.

SVOBODA, D., GRADY, H. J. & HIGGINSON, J. (1966) Aflatoxin $B_1$ injury in rat and monkey liver. *Am. J. Pathol.*, **49**, 1023.

SWENSON, D. H., LIN, J.-K., MILLER, E. C. & MILLER, J. A. (1977) Aflatoxin $B_1$-2,3-oxide as a probable intermediate in the covalent binding of aflatoxin $B_1$ and $B_2$ to rat liver DNA and ribosomal RNA *in vivo*. *Cancer Res.*, **37**, 172.

UNGER, P. D., MEHENDALE, H. M. & HAYES, A. W. (1977) Hepatic uptake and disposition of aflatoxin $B_1$ in isolated perfused rat liver. *Toxicol. Appl. Pharmacol.*, **41**, 523.

VILLA-TREVIÑO, S. & LEAVER, D. D. (1968) Effects of the hepatotoxic agents retrorsine and aflatoxin $B_1$ on hepatic protein synthesis in the rat. *Biochem. J.*, **109**, 87.

WOGAN, G. N. (1968) Biochemical responses to aflatoxins. *Cancer Res.*, **28**, 2282.

WOGAN, G. N. (1969) Metabolism and biochemical effects of aflatoxins. In *Aflatoxin: Scientific Background, Control and Implications.* Ed. Goldblatt. New York: Academic Press. p. 151.

WOGAN, G. N. (1973) Aflatoxin carcinogenesis. In *Methods in Cancer Research*, Vol. 7, Ed. Busch. New York: Academic Press. p. 309.

WOGAN, G. N. (1974) Naturally occurring carcinogens. In *Biology and Biochemistry. The Physiopathology of Cancer, Vol.* 1, Ed. Shubik. Basel: Karger. p. 64.

WOGAN, G. N., EDWARDS, G. S. & SHANK, R. C. (1967) Excretion and tissue distribution of radioactivity from aflatoxin $B_1 - {}^{14}C$ in rats. *Cancer Res.*, **27**, 1729.

WOGAN, G. N. & FRIEDMAN, M. A. (1968) Inhibition by aflatoxin $B_1$ of hydrocortisone induction of rat liver tryptophan pyrrolase and tyrosine transaminase. *Archs Biochem. Biophys.*, **128**, 509.

WOGAN, G. N. & NEWBERNE, P. M. (1967) Dose-response characteristics of aflatoxin $B_1$ carcinogenesis in the rat. *Cancer Res.*, **27**, 2370.

YU, F.-L. (1977) Mechanism of aflatoxin $B_1$ inhibition of rat hepatic nuclear RNA synthesis. *J. Biol. Chem.*, **252**, 3245.